# Tyrosine Phosphorylation of Caspase-8 Abrogates Its Apoptotic Activity and Promotes Activation of c-Src

**DOI:** 10.1371/journal.pone.0153946

**Published:** 2016-04-21

**Authors:** Jennifer LY Tsang, Song Hui Jia, Jean Parodo, Pamela Plant, Monika Lodyga, Emmanuel Charbonney, Katalin Szaszi, Andras Kapus, John C. Marshall

**Affiliations:** 1 Division of Critical Care, Department of Medicine, McMaster University, Hamilton, Ontario, Canada; 2 Division of Critical Care, Department of Medicine, Niagara Health System, Niagara, Ontario, Canada; 3 Keenan Research Centre for Biomedical Science of the Li Ka Shing Knowledge Institute, Toronto, Ontario, Canada; 4 Laboratory of Tissue Repair and Regeneration, Matrix Dynamics Group, Faculty of Dentistry, University of Toronto, Toronto, Ontario, Canada; 5 Department of Medicine, University of Montreal, Montreal, Quebec, Canada; 6 Centre de Recherche de “Hopital du Sacre-Coeur de Montreal, Montreal, Quebec, Canada; 7 Department of Surgery, St. Michael’s Hospital, Toronto, Ontario, Canada; 8 Department of Critical Care Medicine, St. Michael’s Hospital, Toronto, Ontario, Canada; 9 Interdepartmental Division of Critical Care Medicine, University of Toronto, Toronto, Ontario, Canada; Hungarian Academy of Sciences, HUNGARY

## Abstract

Src family tyrosine kinases (SFKs) phosphorylate caspase-8A at tyrosine (Y) 397 resulting in suppression of apoptosis. In addition, the phosphorylation of caspase-8A at other sites including Y465 has been implicated in the regulation of caspase-8 activity. However, the functional consequences of these modifications on caspase-8 processing/activity have not been elucidated. Moreover, various Src substrates are known to act as potent Src regulators, but no such role has been explored for caspase-8. We asked whether the newly identified caspase-8 phosphorylation sites might regulate caspase-8 activation and conversely, whether caspase-8 phosphorylation might affect Src activity. Here we show that Src phosphorylates caspase-8A at multiple tyrosine sites; of these, we have focused on Y397 within the linker region and Y465 within the p12 subunit of caspase-8A. We show that phosphomimetic mutation of caspase-8A at Y465 prevents its cleavage and the subsequent activation of caspase-3 and suppresses apoptosis. Furthermore, simultaneous phosphomimetic mutation of caspase-8A at Y397 and Y465 promotes the phosphorylation of c-Src at Y416 and increases c-Src activity. Finally, we demonstrate that caspase-8 activity prevents its own tyrosine phosphorylation by Src. Together these data reveal that dual phosphorylation converts caspase-8 from a pro-apoptotic to a pro-survival mediator. Specifically, tyrosine phosphorylation by Src renders caspase-8 uncleavable and thereby inactive, and at the same time converts it to a Src activator. This novel dynamic interplay between Src and caspase-8 likely acts as a potent signal-integrating switch directing the cell towards apoptosis or survival.

## Introduction

Caspase-8, the apical caspase in the extrinsic pathway of apoptosis, is activated by the ligation of death receptors [1–4]. Pro-caspase-8 is expressed as a zymogen consisting of an N-terminal pro-domain, with two death effector domains (DED) [5–9], followed by a large (p18) and a small (p12) enzyme subunits. Caspase-8 activation requires its dimerization (in proximity) followed by a 2-step cleavage after aspartic acid (D) residues. Cleavage occurs first after D391 within the linker region and then after D233 at the start of the p18 subunit [1–4,9–12]. Cleavage after D391 is important for the activation of caspase-8 whereas cleavage after D233 allows the release of active caspase-8 from the pro-domain. The mature caspase-8 enzyme, consisting of two p18 and two p12 subunits [5–12], then cleaves and activates downstream proteases including caspase-3, to allow apoptosis to proceed.

Caspase-8 has been shown to be phosphorylated by Src family tyrosine kinases (SFKs) at tyrosine (Y) 397 in caspase-8A [13] (NCBI Nomenclature) and Y380 in caspase-8B (NCBI Nomenclature) [14,15]. We and others have shown that tyrosine phosphorylation of caspase-8 suppresses its pro-apoptotic activity [13,16] and promotes cell migration [15]. Caspase-8 can also be tyrosine phosphorylated following epidermal growth factor (EGF) stimulation [14], which leads to the activation of extracellular signal-regulated kinase (Erk) [17]. Furthermore, tyrosine phosphorylated caspase-8 interacts with c-Src via its Src homology 2 (SH2) domains [15,18] and with the p85 subunit of phosphatidylinositol 3-kinase (PI3K) [19].

Together these data suggest that growth factor signaling could potentially influence caspase-8 activity by promoting its tyrosine phosphorylation. However, the mechanism whereby caspase-8 tyrosine phosphorylation suppresses apoptosis remains to be clarified. Moreover, it is conceivable that tyrosine phosphorylation not only alters the pro-apoptotic function of this caspase but also induces direct pro-survival mechanisms. While such a possibility has been raised by previous studies [17,18], this intriguing question has not been directly addressed.

Here, we show that phosphomimetic mutation of caspase-8A at Y465 suppresses apoptosis by inhibiting caspase-8 cleavage, whereas simultaneous phosphomimetic mutation of caspase-8A at Y397 and Y465 activates c-Src, suggesting that dual phosphorylation converts caspase-8 from a pro-apoptotic to a pro-survival protein that serves as a Src substrate and Src activator.

## Materials and Methods

### Cell Lines

Human embryonic kidney cells (HEK293 cells, CRL-1573, ATCC) and A549 human lung carcinoma cells (CRL-185, ATCC) were cultured in Dulbecco’s modified Eagle’s medium with high glucose (Invitrogen) supplemented with 10% heat inactivated fetal bovine serum and 1% penicillin/streptomycin solution. Chinese Hamster Ovary (CHO cells) were obtained from ATCC (CCL-61) and cultured in α-minimal essential medium with 10% heat inactivated fetal bovine serum and 1% penicillin/streptomycin solution. All cell lines were cultured in a standard humidified incubator at 37°C in a 5% CO_2_ atmosphere.

### Antibodies and Reagents

Antibodies used in these studies were rabbit monoclonal (mAb) anti-active caspase-3 (1:100, Cell Signaling Technology (CST)), rabbit mAb anti-caspase-8 (1:1000, CST), murine mAb anti-green fluorescence protein (GFP) (1:1000, Santa Cruz), murine mAb anti-avian Src (1:500, Millipore), murine mAb anti-Src (1:1000, CST), rabbit mAb anti-phospho-Src (Tyr416) (1:1000, CST), rabbit anti-Erk1/2 (1:1000, CST), rabbit anti-phospho-Erk1/2 (Thr202/Tyr204) (1:1000, CST), mouse mAb anti-phospho-Tyr (clone 4G10) (1:2000, Millipore), murine monoclonal anti-beta-actin (1:4000, Sigma), Cy3-labelled rabbit anti-IgG (1:1000, Jackson Immunoresearch Laboratories), peroxidase-conjugated anti-mouse IgG (1:5000, CST), peroxidase-conjugated anti-mouse light chain specific IgG (1:5000, Jackson Immunoresearch Laboratories) and peroxidase-conjugated anti-rabbit IgG (1:5000, GE Health Care). Caspase-8 inhibitor (Z-IETD-FMK) was purchased from Calbiochem.

### Plasmid Construction

Total RNA from healthy human volunteer neutrophils was extracted using TRIzol reagent, and 1 μg of RNA was transcribed to first-strand cDNA using the Superscript II system (Invitrogen); the resultant cDNA was amplified by PCR using the Expand^™^ High Fidelity PCR System (Roche Molecular Diagnostic) with the following primer set: caspase-8 forward primer (containing a *Hin*dIII site and a Kozak sequence), 5’-GCAAGCTTCGATGGACTTCAGCAGAAATC-3’; caspase-8 reverse primer (containing an *Bam*HI site), 5’-GCGGATCCCAGATCCTCTTCTGAGATGAG-3’. Amplified fragments (caspase-8A or caspase-8B) were cloned into the pEGFP-C1 vector (Invitrogen) according to manufacturer’s instructions. The recombinant plasmids were transfected into DH5α competent cells (Invitrogen), and colonies were identified by restriction enzyme digestion and DNA sequencing (The Centre for Applied Genomics, Hospital for Sick Children, Toronto, Ontario, Canada).

Caspase-8A and caspase-8B point mutations were generated as follows: GFP-caspase-8A or GFP-caspase-8B in pEGFP-C1 backbone was subjected to single rounds of site-directed mutagenesis using QuickChange Site-Directed Mutagenesis kit (Stratagene), according to manufacturer’s recommendations. The primers used are listed in Table 1. After *Dpn*I digestion of the amplified product, the mutant DNA was transfected to XL1-blue supercompetent cells, and colonies identified by restriction enzyme digestion and DNA sequencing (The Centre for Applied Genomics, Hospital for Sick Children, Toronto, Ontario, Canada).

**Table 1:** List of Primers Used for Point Mutations of GFP-Caspase-8 Constructs.

Mutant	Amino Acid Change	Primers Used
Catalytically Inactive	C377S/C360S	5’-ATTCAGGCTAGTCAGGGGG-3’
		5’-CCCCCTGACTAGCCTGAAT-3’
Phosphomimetic at Y397/Y380	Y397E/Y380E	5’-GGAGCAACCCTTTTTAGAAATGG-3’
		5’-CCATTTCTAAAAAGGGTTGCTCC-3’
Non-Phosphorylatable at Y397/Y380	Y397F/Y380F	5’-GGAGCAACCCGAGTTAGAAATGG-3’
		5’-CCATTTCTAACTCGGGTTGCTCC-3’
Phosphomimetic at Y465/Y448	Y465E/Y448E	5’-GAAGTGAACGAGGAAGTAAGC-3’
		5’-GCTTACTTCCTCGTTCACTTC-3’
Non-phosphorylatable at Y465/Y448	Y465F/Y448F	5’-GAAGTGAACTTTGAAGTAAGC-3’
		5’-GCTTACTTCAAAGTTCACTTC-3’

All of the final constructs were verified by DNA sequencing (The Centre for Applied Genomics, Hospital for Sick Children, Toronto, Ontario, Canada).

We generated double mutant (Y397F+Y465E) caspase-8A by performing mutagenesis from the Y397F construct with Y465E primers and double mutant (Y397E+Y465E) caspase-8 by performing mutagenesis from Y397E construct with Y465E primers. We also generated double mutant (Y397E+Y465F) caspase-8A by performing mutagenesis from the Y397E construct with Y465F primers and double mutant (Y397F+Y465F) caspaes-8 by performing mutagenesis from the Y397F construct with Y465F primers.

Constitutively active chicken Src (Y527F Src) in pCMV1 vector was kindly provided by Dr. D. Flynn (West Virginia University).

### Transfection of Cell Lines

The above plasmids, GFP-caspase-8A or 8B (wild type or mutants) with or without Y527F Src, were transfected into HEK293 cells, A549 cells or CHO cells. We transfected 0.5 μg or 3 μg of each plasmid into 80–90% confluent cells in 12-well plates or 10-cm dishes (coated with poly-D-Lysine (Sigma)) respectively using 3 μL or 18 μL of ExtremeGENE 9 reagent (Roche Molecular Diagnostic) for 24 hours according to the manufacturer’s instructions. Transfection efficiency was approximately 30–40%.

For the caspase-8 inhibitor study, cells were transfected with appropriate plasmids for 9 hours at 37°C, then treated with 20 μM of caspase-8 inhibitor (Z-IETD-FMK) (Calbiochem) and incubated for 15 hours at 37°C.

### Immunoprecipitation and Western Blotting

For immunoprecipitation studies of exogenous GFP-caspase-8 or avian Y527F Src from HEK293, A549 or CHO cells, confluent HEK293, A549 or CHO cells grown in 10-cm dishes were lysed for 10 minutes on ice in Triton X-100 lysis buffer (10 mM Tris, pH 7.4, 150 mM NaCl, 5 mM EDTA, 1% Triton X-100, 10 mM NaF) with Complete Mini EDTA Free Protease Inhibitor Cocktail (Roche Molecular Diagnostic), PhosSTOP (Roche Molecular Diagnostic), 1 mM phenylmethylsulfonyl fluoride and 1 mM sodium vanadate. We then centrifuged lysates at 4°C for 10 min at 12 000 rpm. Supernatants were pre-cleared with Pierce Protein G Agarose (Thermo Scientific) for one hour at 4°C, then centrifuged at 4°C for 1 minute at 12 000 rpm to remove agarose beads. Protein concentration in lysates was measured using the BCA protein assay (Thermo Scientific), then 2 μg of murine monoclonal GFP antibody (Santa Cruz) or murine monoclonal avian Src antibody (Millipore) was added to 4 mg of protein and lysates incubated for 1 hour at 4°C. Then 25 μL of Pierce Protein G Agarose (Thermo Scientific) was added and lysate incubated for a further hour. Cell lysates were centrifuged and protein G agarose beads were washed three times in Triton X-100 buffer, then boiled in Laemmli buffer for 5 minutes prior to SDS-PAGE and Western analysis.

For Western blot analysis, we lyzed confluent wells of 12-well plate of HEK293, A549 or CHO cells for 10 minutes on ice in Triton X-100 lysis buffer (10 mM Tris, pH 7.4, 150 mM NaCl, 5 mM EDTA, 1% Triton X-100, 10 mM NaF) with Complete Mini EDTA Free Protease Inhibitor Cocktail (Roche Molecular Diagnostic), PhosSTOP (Roche Molecular Diagnostic), 1 mM phenylmethylsulfonyl fluoride and 1 mM sodium vanadate. We then boiled lysates in Laemmli buffer for 5 minutes prior to SDS-PAGE and Western analysis.

### Mass Spectrometry

Immunoprecipitated GFP-caspase-8A (C377S or Y465E) was resolved on SDS-PAGE and bands corresponding to GFP-caspase-8A were excised from the gel. The samples were submitted to the Mass Spectrometry Facility of the Hospital for Sick Children in Toronto, Ontario for in-gel trypsin digestion and liquid chromatography tandem mass spectrometry (LC-MS/MS) analysis.

### Microscopy and Immunofluorescence Analysis

For immunofluorescence staining, cells were grown on glass coverslips coated with poly-D-Lysine (Sigma). Following transfection, cells were rapidly fixed by 4% paraformaldehyde for 30 minutes and washed with PBS. After quenching the paraformaldehyde with 100 mM glycine in PBS, the cells were permeabilized with 0.1% Triton-X-100 in 1% (w/v) albumin for 20 minutes and blocked with 3% (w/v) albumin for 1 hour. The coverslips were then incubated with primary antibody (anti-active-caspase-3) for 1 hour, washed and incubated with secondary antibody (Cy3-labelled rabbit anti-IgG) for 1 hour. The coverslips were washed with PBS and mounted on glass slides using fluorescence mounting medium (Dako). The staining was visualized using an Olympus 1X81 microscope (Mellville) coupled to an Evolution QEi Monochrome camera controlled by the MetaMorph software. The percentage of GFP-positive cells (GFP-caspase-8 transfected cells) that were also Cy3 positive (active caspase-3 positive) was quantified by counting the number of double GFP and Cy3 positive cells and divided by the number of single GFP positive cells by counting a minimum of 200 GFP positive cells in each experiment.

### Quantification of Apoptosis

We measured rates of apoptosis by flow cytometry, quantifying the uptake of propidium iodide in Triton X-100 permeabilized cells as previously described [20]. Briefly, Triton X-100-permeabilized cells were incubated with propidium iodide (50 μg/mL) and analyzed using a Coulter Epics XL-MCL cytoflurometer (Hialeah, FL). A minimum of 5000 events was collected and analyzed.

### Statistical Analysis

Statistical significance was determined using the Student t-test, Wilcoxon Signed Rank Test or Kruskal Wallis Test with Dunn’s Multiple Comparison Test, or One way ANOVA when there were more than two samples for comparison. GraphPad Prism 4.0 software was used.

## Results

### Tyrosine phosphorylation of caspase-8A in multiple sites follows Src expression

**Fig 1:**
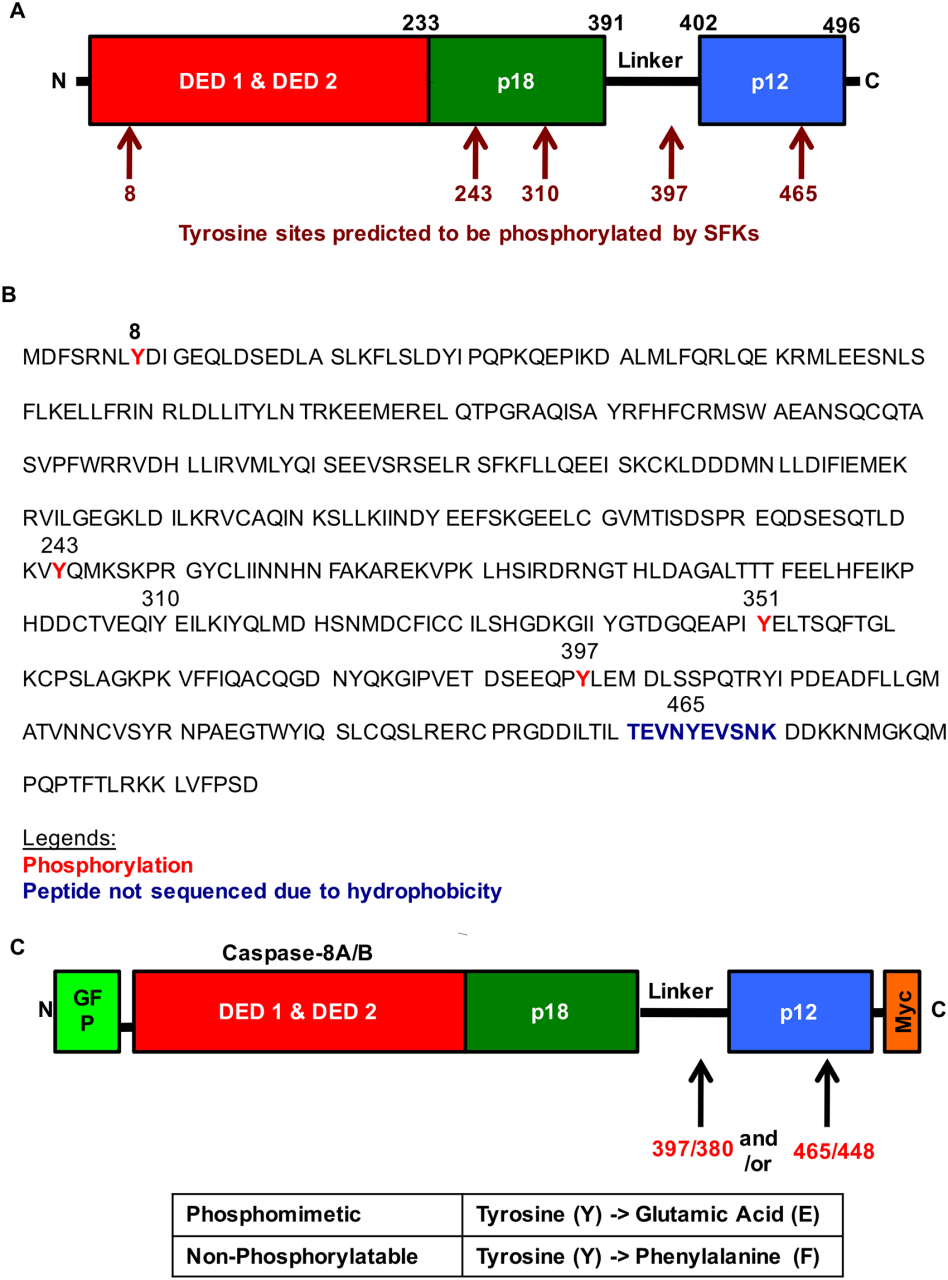
Tyrosine phosphorylation of caspase-8A in multiple sites follows Src expression. A) Caspase-8A structure and SFK tyrosine phosphorylation sites predicted by GPS2.1. B) HEK293 cells were co-transfected with inactive C377S mutant of GFP-caspase-8A and Y527F Src for 24 hours then lysates immunoprecipitated using an anti-GFP antibody. GFP-caspase-8A IP was subjected to SDS-PAGE, and the appropriate band (80 kDa) was excised and sent for LC-MS/MS analysis. C) Mutagenesis was performed at Y397/Y380 and Y465/Y448 sites to generate phosphomimetic (tyrosine to glutamic acid) and non-phosphorylatable (tyrosine to phenylalanine) caspase-8A/B mutants.

While previous studies have shown that SFKs could tyrosine phosphorylate caspase-8A (NCBI nomenclature) at Y397 [13] or caspase-8B (NCBI nomenclature) at Y380 [14,15], these are not the only potential Src target residues in caspase-8. To identify further potential Src target sites, we used a SFK phosphorylation prediction tool, GPS 2.1 [21]. We showed that in caspase-8A, Y8 within the N-terminal domain; Y243, Y310 and Y351 within the p18 subunit; Y397 within the linker region and Y465 within the p12 subunit could be phosphorylated by SFKs (Table 2 and Fig 1A).

**Table 2:** Predicted SFK tyrosine phosphorylation sites in caspase-8.

Amino Acid Position	Peptide Sequence
8	NDFSRBK**Y**DUGEQKD
243	SQTLDKV**Y**QMKSKPR
310	DCTVEQI**Y**EILKIYQ
397	TDSEEQP**Y**LEMDLSS
465	TILTEVN**Y**EVSNKDD

Using mass spectrometry, we were able to verify that phosphorylation of caspase-8A at Y8, Y243, Y351 and Y397 follows c-Src expression in HEK293 cells (Fig 1B). The sequence coverage was only approximately 50%, and the hydrophobic peptide region surrounding Y465 was not released from the SDS-PAGE gel precluding the direct confirmation of phosphorylation at this target site.

Since the p12 subunit of caspase-8 is crucial for its dimerization and subsequent enzymatic activation, we examined the functional consequences of the phosphorylation of the Y465 residue, which is located in the p12 subunit. Knowing that Y397, located in the linker region, participates in caspase-8 regulation, we also sought to determine the functional consequences of Y397 phosphorylation alone or in combination with Y465 phosphorylation. To assess the functional relevance of these sites, we generated phosphomimetic and non-phosphorylatable caspase-8A mutants at Y397 and Y465 residues and caspase-8B mutants at Y380 and Y448, labeling them with GFP in the N-terminus and c-Myc at the C-terminus allowing direct visualization of transfected cells using fluorescence microscopy or detecting the expressed molecules and their fragments by Westerns blotting using antibodies against the tags (Fig 1C).

### Y465 phosphomimetic mutation inhibits caspase-8A cleavage

Although tyrosine phosphorylation of caspase-8 has been shown to reduce its activity, the underlying mechanism has not been fully elucidated. To investigate the mechanism whereby tyrosine phosphorylation suppresses caspase-8 enzymatic activity, we examined the cleavage pattern of wild type (WT) caspase-8 and caspase-8 phosphomutants. HEK293 cells were transfected with GFP-caspase-8A constructs and analyzed by Western blotting. We probed the blots both with anti-GFP antibody, which recognizes the N-terminus of the heterologously expressed caspase-8 and with anti-caspase-8 antibody, which recognizes the C-terminus (p12 subunit) of both the endogenous and the heterologously expressed caspase-8 protein.

**Fig 2:**
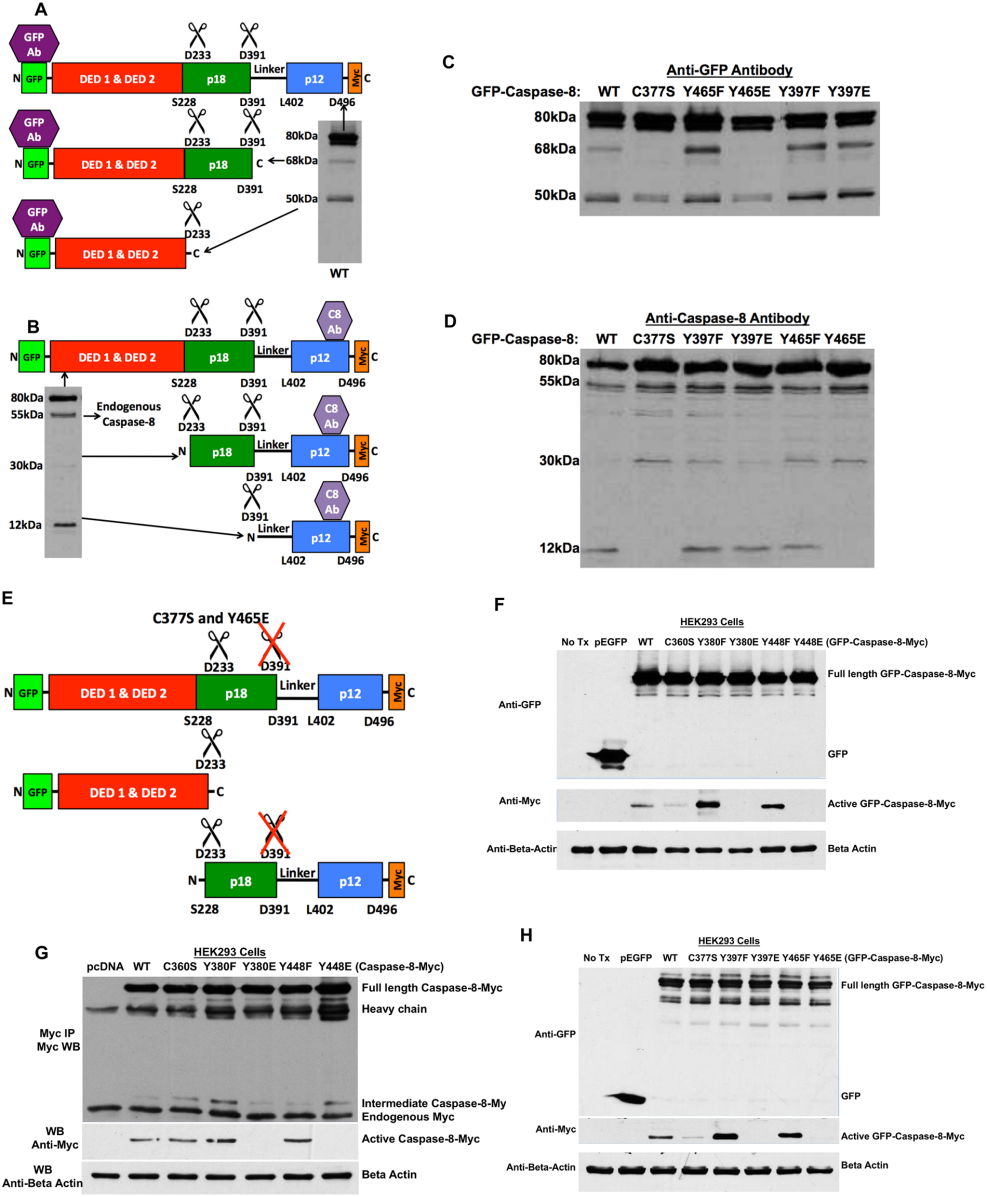
Y465 phosphomimetic mutation inhibits caspase-8A cleavage. A) Upon activation, caspase-8 is autocatalytically cleaved first after D391, and then after D233. Using an anti-GFP antibody that binds to the N-terminus of the molecule, a 68-kDa fragment is visualized when GFP-caspase-8A is cleaved after D391, whereas a 50-kDa fragment is visualized when GFP-caspase-8A is cleaved after D233. B) Using an anti-caspase-8 antibody that binds to the C-terminus of the molecule, a 30-kDa fragment is visualized when GFP-caspase-8A is cleaved after D233, whereas a 12-kDa fragment is visualized when GFP-caspase-8A is cleaved after D391. A 55-kDa band represents endogenous caspase-8. C) HEK293 cells were transfected with GFP-caspase-8A WT or mutants for 24 hours. Whole cell lysates were subjected to Western blot analysis with anti-GFP antibody that recognizes the N-terminal end of GFP-caspase-8A. D) Whole cell lysates were subjected to Western blot analysis with anti-caspase-8 antibody that recognizes the C-terminal end of GFP-caspase-8A. E) Both the inactive C377S and Y465E GFP-caspase-8A mutants failed to undergo cleavage at D391, the essential step of the activation of caspase-8. F-H) HEK293 cells were transfected with GFP-caspase-8B (F), Myc-caspase-8B (G), GFP-caspase-8A (H) WT or mutants for 24 hours. Whole cell lysates were subjected to Western blot analysis with anti-GFP antibody that recognizes the N-terminal end of GFP-caspase-8 and anti-Myc antibody that recognizes the C-terminal end of GFP-caspase-8 (active caspase-8).

We verified that WT caspase-8 could be cleaved after D391, resulting in a 68-kDa N-terminal fragment; and after D233, resulting in a 50-kDa N-terminal fragment (Fig 2A). In accordance with this, anti-caspase-8 antibody visualized the full-length protein as well as 30-kDa and 12-kDa C-terminal fragment corresponding to cleavage after D233 and D391 respectively (Fig 2B). Similar cleavage patterns were observed in Y465F, Y397F and Y397E mutants, indicating that these mutations do not alter the processing of caspase-8A. However, similar to the C377S inactive mutant, Y465E (phosphomimetic) mutant was not cleaved after D391, as indicated by the fact that it failed to produce a 68-kDa N-terminal fragment and a 12-kDa C-terminal fragment (Fig 2C and 2D). Together, these data imply that phosphomimetic modification of Y465 prevents its cleavage after D391 (Fig 2E).

While Y465E proves to be uncleavable in all experiments (verified with caspase-8B constructs with (Fig 2F) or without (Fig 2G) GFP-tag, the Y397E showed variability in its ability to be cleaved (Fig 2F, 2G and 2H). In certain experiments, we noted cleavage in Y397E mutant and in some experiments, we did not observe cleavage in Y397E mutant.

The reason for this variability is unknown, but it may be dependent upon the phosphorylation state of Y465 residue.

### Y465 phosphomimetic modification of caspase-8A prevents caspase-3 activation and apoptosis

**Fig 3:**
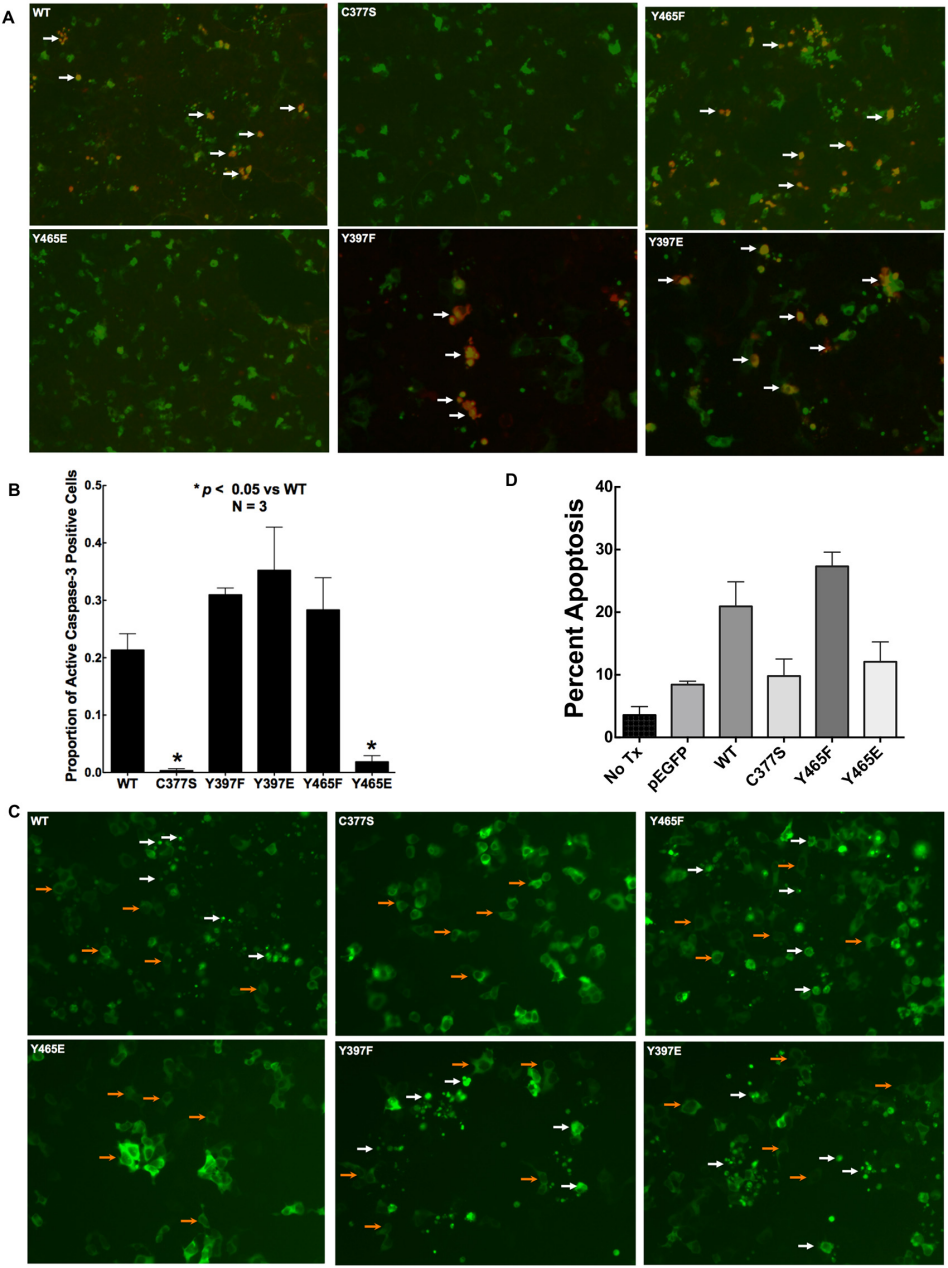
Y465 phosphomimetic modification of caspase-8A abolishes its ability to activate caspase-3 and to induce apoptosis. WT GFP-caspase-8A mutants were transfected into HEK293 cells. A) Cells were fixed and immunostained with anti-cleaved caspase-3 antibody. Green represents GFP-caspase-8A expressing cells; orange represents active caspase-3 in GFP-caspase-8A expressing cells (white arrow). B) The percentage of active caspase-3 positive cells is displayed. N = 3, * *p* < 0.05. C) Cell morphology of transfected cells was examined using live fluorescence microscopy. Healthy cells are identified with orange arrows whereas apoptotic cells characterized by cell rounding or apoptotic bodies are identified with white arrows. D) Transfected cells were permeabilized and stained with propidium iodide to qualified the percentage of cells expressing hypodiploid DNA representing DNA fragmentation seen in apoptosis. N = 3, *p* < 0.05.

To assess the apoptotic activity of these caspase-8A mutants, we first transfected WT and mutant GFP-caspase-8A into HEK293 cells and detected active (cleaved) caspase-3 by immunofluorescence microscopy (Fig 3A). After 24 hours of transfection, approximately 20% of the WT GFP-caspase-8A-expressing cells were positive for active caspase-3 (Fig 3B). Y397F, Y397E and Y465F mutants resulted in caspase-3 cleavage in 30–35% of the transfected cells. As expected, fewer than 1% of the cells expressing C377S exhibited active caspase-3 staining. More importantly, Y465E overexpression also failed to induce caspase-3 activation as fewer than 1% of the cells expressing Y465E exhibited active caspase-3 staining.

In accordance with these findings, transfection with WT GFP-caspase-8A led to significant changes in cell morphology with cell rounding and the formation of apoptotic bodies [22], consistent with the ensuing apoptosis; similar changes were seen following transfection with the Y397F, Y397E and Y465F mutants. However, both C377S and Y465E mutants failed to cause these changes (Fig 3C), confirming that these mutations led to the loss of the apoptotic activity of casapse-8. To substantiate the critical role of Y465E in apoptosis induction, we performed an alternative apoptosis assay using propidium staining to detect the formation of hypodiploid DNA, which confirmed the morphological data (Fig 3D). Moreover, we have previously found that Y465E reduced apoptosis in HL-60 cells [13].

### Y465 phosphomimetic caspase-8A promotes Src activation

Several Src substrates, including focal adhesion kinase (FAK) [23], actin filament-associated protein (AFAP-110) [24] and XB-130 [25], can also act as Src activators by stabilizing the open (active) conformation of the kinase. Thus we asked whether tyrosine phosphorylation of caspase-8 enables it to regulate Src activity. Src activation involves the dephosphorylation of an inhibitory tyrosine residue (Y527) [26,27] and the autophosphorylation of an activating loop tyrosine residue (Y416) [28–30]. To elicit Src-mediated tyrosine phosphorylation, we used a constitutively active Src (Y527F), and to simultaneously monitor the ensuing (additional) Src activation, we followed the phosphorylation of Y416. As additional measures of Src activation, we evaluated global protein tyrosine phosphorylation and Erk1/2 phosphorylation, a downstream consequence of Src activation.

**Fig 4:**
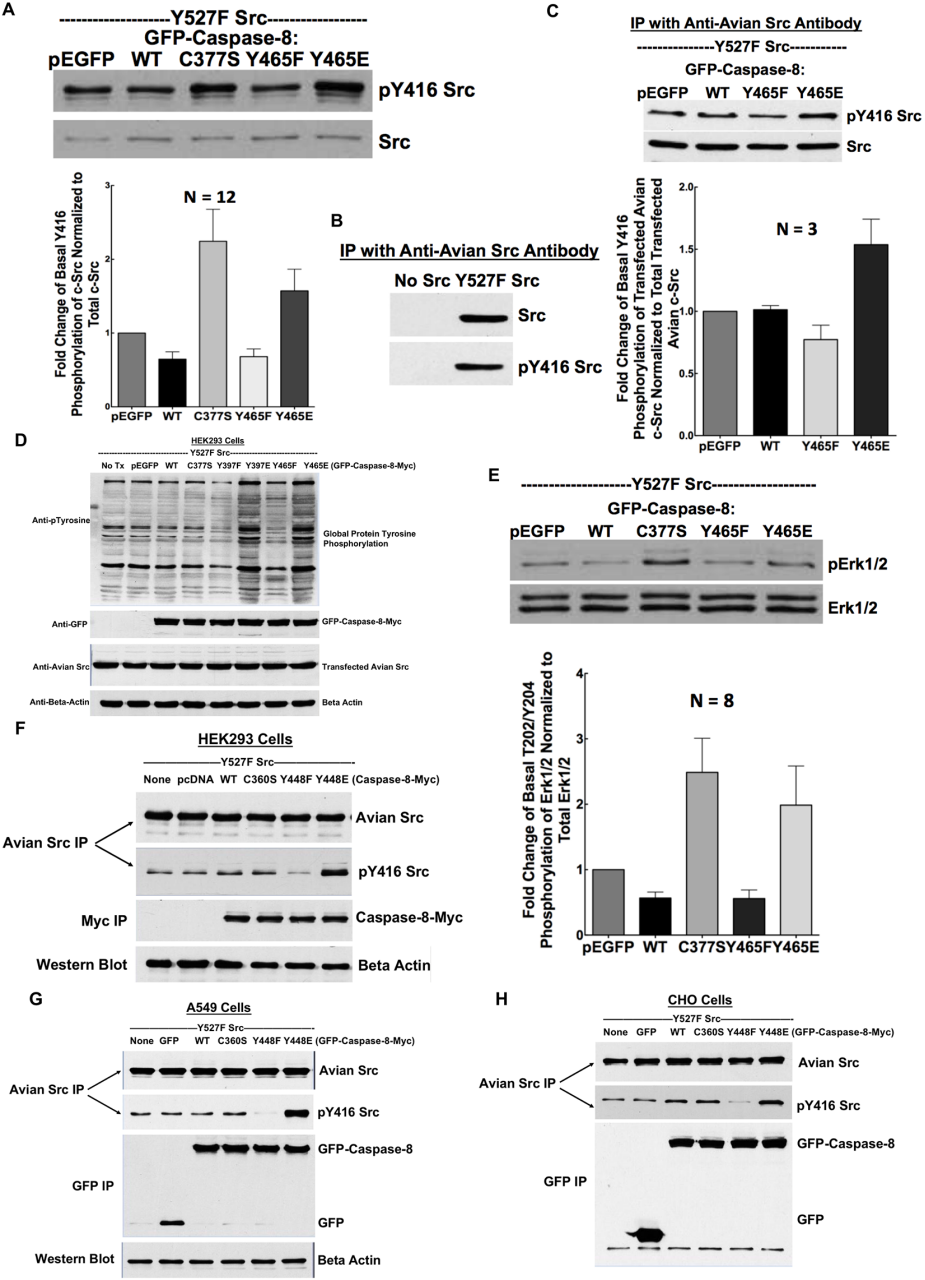
Y465 phosphomimetic modification of caspase-8A promotes Src activation and activity. HEK293 cells were co-transfected with empty GFP plasmid or GFP-tagged caspase-8A (WT and various mutants) and Y527F Src for 24 hours. A) Whole cell lysates were subjected to Western blot analysis with antibodies to pY416 Src and Src. Src Y416 phosphorylation was normalized to total Src. N = 12, *p* < 0.0001. B) HEK293 cells were co-transfected with GFP-caspase-8A with or without Y527F Src for 24 hours. Y527F Src was immunoprecipitated using an anti-avian Src antibody and probed with anti-avian Src and anti-pY416 Src antibody to demonstrate specificity of anti-avian Src antibody. C) HEK293 cells were co-transfected with empty GFP plasmid or GFP-caspase-8A (WT and various mutants) with Y527F Src for 24 hours; Y527F Src was immunoprecipitated using an anti-avian Src antibody then probed with anti-avian Src and anti-pY416 Src antibodies. Y416 phosphorylation signal of avian Src was normalized to total expressed avian Src level. N = 3, *p* = 0.02. D) Whole cell lysates were subjected to Western blot analysis with anti-phospho-tyrosine antibody to show global protein tyrosine phosphorylation. The same blot was also probed with anti-GFP antibody to look at the expression of transfected GFP-caspase-8A; anti-avian Src antibody to look at the expression of transfected avian Src; and anti-beta actin antibody to look at the expression of beta actin as a loading control. E) Whole cell lysates were subjected to Western blot analysis to evaluate Erk1/2 phosphorylation. Phosphorylation of Erk1/2 was normalized to total expressed Erk1/2 level. N = 8, *p* < 0.001. F-H) HEK293 (F), A549 (G) and CHO (H) cells were co-transfected with empty GFP plasmid or GFP-tagged caspase-8B (WT and various mutants) with Y527F Src for 24 hours; Y527F Src was immunoprecipated using anti-avian Src antibody. Then precipitates were immunoblotted with antibodies to pY416 Src and avian Src.

HEK293 cells were co-transfected with Y527F Src and pEGFP empty plasmid or GFP fusion plasmid encoding for WT or mutant GFP-caspase-8A constructs—C377S (inactive), Y465E (phosphomimetic), or Y465F (non-phosphorylatable) mutants. Compared to empty plasmid, both the Y465 phosphomimetic mutant and the C377S inactive caspase-8A mutant increased the phosphorylation of Y416 of Src (Fig 4A).

Since only a fraction of HEK293 cells (approximately 30%) expressed the transfected caspase-8A mutants, we took advantage of the fact that the transfected Y527F Src mutant was of chicken origin and could be immunoprecipitated with a chicken Src-specific antibody (Fig 4B). This allowed us to specifically measure the activation of transfected Src. Using this approach, we confirmed that Y465E GFP-caspase-8A mutant augmented Y416 phosphorylation of Src (Fig 4C).

In keeping with these data, both the inactive C377S mutant and the Y465 phosphomimetic mutant of caspase-8A increased global protein tyrosine phosphorylation (Fig 4D). Moreover, the inactive C377S and Y465E GFP-caspase-8A mutants promoted Erk1/2 phosphorylation (Fig 4E).

Caspase-8 is expressed in different isoforms. In order to show generalizability of caspase-8 (Y465E in caspase-8A isoform)-dependent Src activation, we performed co-transfection experiment in HEK293 cells with Y527F Src and pEGFP empty plasmid or GFP fusion plasmid encoding for WT or mutant GFP-caspase-8B constructs—C360S (inactive), Y448E (phosphomimetic), or Y448F (non-phosphorylatable) mutants. We showed similar results as with caspase-8A isoform that Y448E (equivalent to Y465E in caspase-8A) has the capacity to induce Src activation (Fig 4F)

To further show generalizability of caspase-8A/B (Y465E/Y448E)-dependent Src activation, we performed double (caspase-8B and Y527FSrc) transfection experiments in A549 cells (Fig 4G) and in Chinese Hamster Ovary (CHO) cells (Fig 4H) and showed similar results.

### Phosphomimetic modification of both Y397 and Y465 are necessary but neither alone is sufficient for Src activation

**Fig 5:**
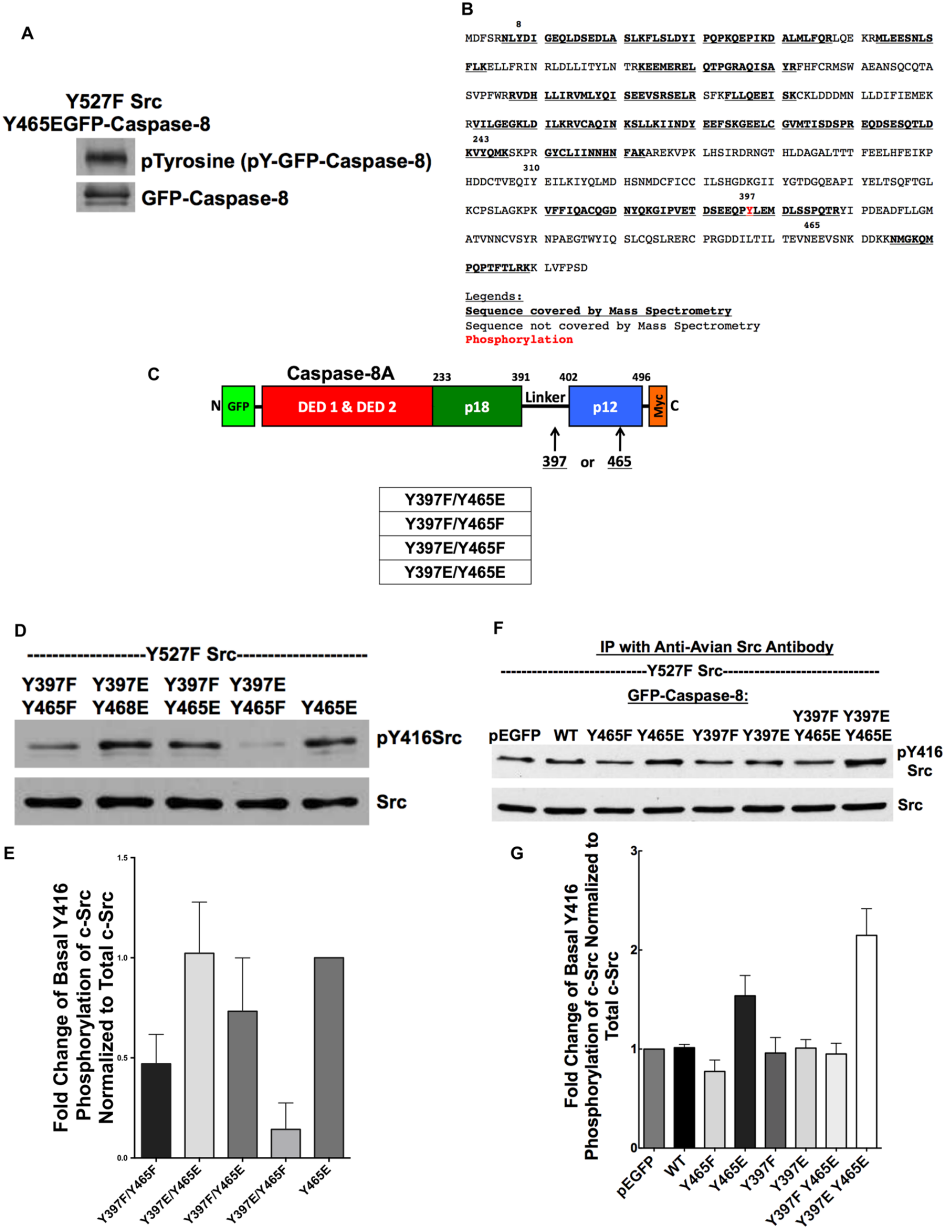
Y465 phosphomimetic modification of caspase-8A is necessary but not sufficient for Src activation. A) HEK293 cells were co-transfected with Y465E GFP-caspase-8A mutant and Y527F Src for 24 hours. Whole cell lysates were subjected to Western blot analysis with an anti-phosphotyrosine antibody (non-reactive to glutamic acid residue in phosphomimetic mutant) and an anti-GFP antibody. Y465E of GFP-caspase-8A was tyrosine phosphorylated. B) HEK293 cells were co-transfected with Y465E GFP-caspase-8A mutant and Y527F Src for 24 hours. GFP-caspase-8A IP was sent for LC-MS/MS analysis. C) This is a schematic figure showing the location of tyrosine residues that we mutated. D, E) We transfected HEK293 cells with Y527F Src and various GFP-caspase-8A mutants for 24 hours. Whole cell lysates were subjected to Western blot analysis with pY416Src antibody and Src antibody. N = 4, *p* = 0.001 (Y465E vs Y397E/Y465F); *p* = 0.139 (Y465E vs Y397F/Y465E). F, G) We transfected HEK293 cells with Y527F Src and pEGFP empty plasmid or pEGFP fusion plasmid with WT or various mutants of GFP-caspase-8 for 24 hours. We then immunoprecipitated transfected avian Src and resolve the immunoprecipitate on SDS-PAGE for Western blot analysis. N = 3, *p* = 0.04.

When Y465E mutant caspase-8A was co-transfected with Y527F Src, the mutant caspase-8A exhibited substantial tyrosine phosphorylation suggesting that tyrosine residues other than Y465 became highly phosphorylated (Fig 5A). Of note, phosphotyrosine antibody does not react to the glutamic acid residue of phosphomimetic caspase-8A mutant. Since we have shown that Src overexpression led to Y397 phosphorylation (Fig 1B) and Y397 is located in the SH2 domain binding phosphotyrosine motif, we asked whether phosphorylation at Y397 is also important (either as a prerequisite or as a contributor) for caspase-8-dependent Src activation as observed in cells transfected with Y465E mutant.

To verify that the Y465E construct is indeed phosphorylated at Y397, we co-transfected HEK293 cells with Y465E GFP-caspase-8A mutant and Y527F Src for 24 hours, then immunoprecipitated transfected GFP-caspase-8A using an anti-GFP antibody. The GFP-caspase-8A IP was resolved on SDS-PAGE, and subjected in-gel trypsin fragments to liquid chromatography-tandem mass spectrometry (LC-MS/MS) analysis. We found that in Y465E mutant caspase-8A (Fig 5A), Y397 was phosphorylated by Src (Fig 5B).

To address the potential contribution of Y397 phosphorylation to the activation of Src, we generated double mutants—Y397E + Y465F, Y397F + Y465F, Y397F+Y465E and Y397E+Y465E (Fig 5C). We then transfected HEK cells with these casapase-8A mutants along with Y527F Src and probed whole cell lysates for pY416 Src (Fig 5D and 5E). While the double E mutant as well as Y465E caused marked phosphorylation of Src, Y397F/465E appeared to exhibit less (or marginal) activation. Interestingly, Y397E/Y465F failed to cause any activation (in fact seemed to have an inhibitory effect), suggesting, that in the absence of Y465 phosphorylation, Y397 phosphorylation alone is unable to trigger significant Src activation. While suggestive, the interpretation of these experiments is hampered by the fact that whole cell lysates were analyzed, and thus the pY416 signals emanated both from transfected and not-transfected Src, although only fraction of cells expressed Y527F Src and the indicated caspase-8A mutants. To overcome this confounding factor, we immunoprecipitated the transfected avian Src and quantified its phosphorylation upon transfection with casapse-8A constructs harboring single or double modification(s) at the investigated sites (Fig 5F and 5G). The results indicate that the single Y465E mutant is capable of Src activation (as opposed to the singe Y397E mutant), while Y397F/Y465E is not. Further, the Y397E/Y465E double phosphomimetic mutant exhibits stronger Src activating capacity than Y465E. Considered together the most plausible interpretation of these data is that phosphomimetic modification of Y465 is a requisite for Src activation, and phosphorylation of Y397, while not sufficient alone, has a permissive and potentiating role in the process. It is conceivable that the fact that Y465E alone is sufficient reflects the possibility that a fraction of Y465E molecules are spontaneously phosphorylated at Y397, a scenario supported by our mass spectrometry data. In any case our finding clearly showed that Src can be activated by tyrosine phosphorylated casapase-8A and modification of Y465 plays a key role in this process.

Certain Src substrates also act as potent regulators of Src. Moreover, caspase-3 activation was proposed to inhibit Fyn, another Src family kinase [31]. These findings thus raised the possibility of a mutual regulation between caspase-8 and Src. Having shown that phosphorylation of caspase-8 not only inhibits its apoptotic activity, but also promotes Src activation, we asked whether active pro-apoptotic caspase-8 could suppress its own tyrosine phosphorylation by Src.

**Fig 6:**
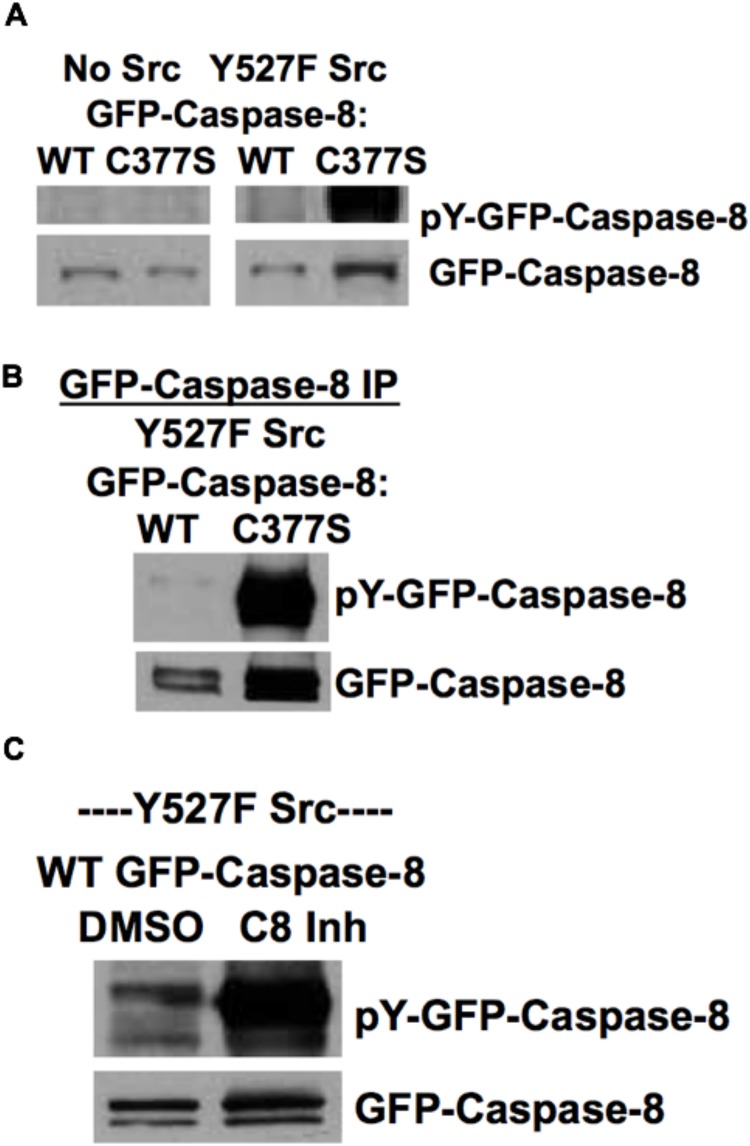
Inhibition of caspase-8 activity enhances Src-dependent tyrosine phosphorylation of caspase-8. A) HEK293 cells were transfected with GFP-caspase-8A (WT or C377S inactive mutant) with or without Y527F Src for 24 hours. Whole cell lysates were subjected to Western blot analysis with anti-phospho-tyrosine antibody and anti-GFP antibody. B) HEK293 cells were transfected with GFP-caspase-8A (WT or C377S inactive mutant) with Y527F Src for 24 hours. GFP-caspase-8A was immunoprecipitated with anti-GFP antibody and precipitates were probed with anti-phospho-tyrosine antibody and anti-GFP antibody. C) HEK293 cells were transfected with WT GFP-caspase-8A and Y527F Src for 9 hours followed by treatment with DMSO vehicle control or caspase-8 inhibitor (20 μM) for 15 hours. Whole cell lysates were subjected to Western blot analysis with anti-phospho-tyrosine antibody and anti-GFP antibody.

To address this question, we transfected HEK293 cells with WT GFP-caspase-8A or C377S catalytically inactive GFP-caspase-8A with or without Y527F Src, and used Western blot analysis of whole cell lysates to confirm that tyrosine phosphorylation of GFP-caspase-8A follows Src expression. In the absence of Y527F Src, there was no tyrosine phosphorylation at the molecular weight corresponding to GFP-caspase-8A. However, transfection with Y527F Src resulted in a faint phosphotyrosine signal in the WT GFP-caspase-8A transfected cells and a much stronger response in the C377S inactive GFP-caspase-8A transfected cells (Fig 6A). To verify the molecular identity of the phosphorylated bands, we transfected HEK293 cells with Y527F Src and WT or C377S GFP-caspase-8A, then immunoprecipitated GFP-caspase-8A using an anti-GFP antibody and immunoblotted with anti-phosphotyrosine antibody (Fig 6B). This confirmed that caspase-8 tyrosine phosphorylation signal was much stronger in the C377S caspase-8A mutant compared to WT caspase-8A.

Caspase-8 activation requires dimerization followed by cleavage. The dissociation constant (K_D_) for homodimerization of caspase-8 is in the low micromolar range [27,32,33]. Since the intracellular concentration of caspase-8 is in the nanomolar range, in order to dimerize, it is necessary that caspase-8 molecules be brought into close proximity by the binding of the DED to the DED of adaptor molecular complexes such as the death inducing signaling complex (DISC) [6]. However, when caspase-8 is overexpressed by transfection, the intracellular concentration might exceed the K_D_, potentially promoting activation of caspase-8 by induced proximity.

We asked whether overexpression of caspase-8 (which likely results in caspase-8 activation by proximity-induced dimerization) could suppress its tyrosine phosphorylation by Src. To this end we transfected HEK293 cells with Y527F Src and WT GFP-caspase-8A and then incubated the transfected cells with or without caspase-8 inhibitor. In the presence of caspase-8 inhibitor, the GFP-caspase-8A tyrosine phosphorylation signal was much stronger (Fig 6C), suggesting that Src-dependent tyrosine phosphorylation of caspase-8 was enhanced in the absence of caspase-8 activity. In other words, activated caspase-8 suppresses its own tyrosine phosphorylation by Src. This may also explain why in the presence of transfected Src, the transfected wild type caspase-8 did not lead to further Src activation.

## Discussion

Caspase-8 has multiple functions independent of apoptosis, including the regulation of inflammation [22], cell proliferation, migration [19,34], immune cell activation [35–37], endosome trafficking [38,39], and NF-κB activation [40,41]. Recent data suggest that deletion or silencing of caspase-8 gene occurs extremely infrequently in cancers [42], while *increased* expression of caspase-8 has been documented in lung cancers [43]. Moreover, a significant fraction of aggressive stage IV neuroblastoma cells (10–30%) maintain caspase-8 expression, and inactivation mutations are surprisingly rare [44–47]. Together, these observations raise the possibility that caspase-8 may contribute to certain aspects of carcinogenesis. More intriguingly, tyrosine phosphorylation of caspase-8 has also been shown in colon cancer cells [14], suggesting that post-translational modification of caspase-8 protein may play a role in the pathogenesis of cancer development and progression.

SFKs, in particular Src, have been shown to promote mitogenesis, cell growth and survival [48–51]. Src is also involved in tumorigenesis and tumor progression by promoting cell growth and cell migration [52–60]. Moreover, a naturally occurring Src mutation (truncated at amino acid 531) is seen in both advanced colon cancer and endometrial carcinoma cells. The truncated form of Src has increased Src activity because the autoinhibitory Y530 (Y527 in avian Src) fails to interact with its own SH2 domain, therefore preventing it from staying in an inactive close conformation [61,62]. This naturally occurring truncation mutant of Src is similar to our Y527F constitutively active Src. We [13] and others [14,15] have shown that SFKs could tyrosine phosphorylate caspase-8 which in turn alters its activity and impacting downstream apoptotic pathway.

Our study reports that phosphomimetic modification of caspase-8A at Y465 prevented the cleavage and subsequent activation of caspase-3 and the induction of apoptosis. While previous studies [13–15], proposed that phosphomimetic modification at Y397 prevents apoptosis, we find that this modification alone may not be sufficient to consistently abrogate apoptosis or promote survival. We propose that the variability of the anti-apoptotic impact of Y397E could be explained by the concomitant phosphorylation status of Y465 residue in any particular experiment. Our data are consistent with the notion that efficient inactivation of caspase-8 and prevention of apoptosis requires phosphorylation both at Y397 and Y465.

Caspase-8 dimerization requires threonine (T) 484 and phenylalanine (F) 485 [27]. Y465 is located in α-helix five, whereas T484 and F485 are located in β-sheet six. α-helix five and β-sheet six are in close proximity in the three-dimensional structure of the molecule, and our results suggest that a negative charge at residue 465 might distort the three-dimensional structure of the caspase-8 molecule near the dimerization interface, thereby interfering with the interaction of the monomers. Such a block in dimerization would result in the loss of caspase-8 cleavage and activation. This would also explain the lack of apoptotic effects seen in Y465 phosphomimetic mutant of caspase-8A.

We further demonstrated that phosphomimetic modification of Y465 of caspase-8A not only prevents apoptosis, but actively promotes cell survival through enhancement of Src activation. Moreover, we found that the phosphomimetic modification of Y465 is necessary for the activation of Src, while Y397 plays a permissive and potentiating effect in this process. Phosphorylation of Y397 provides a phosphotyrosine peptide (pY_397_LEM) that could bind to the SH2 domain of Src, maintaining the Src molecule in an open conformation, thereby allowing autophosphorylation at Y416 of Src, and its consequent increase in kinase activity towards other substrates. We have previously shown that the tyrosine phosphorylated caspase-8 can bind to Lyn [13], while others have demonstrated that in the presence of constitutively active (Y527F) Src, caspase-8B interacted with the Src SH2 domain. However, when caspase-8B harbored a non-phosphorylatable modification at Y380 (equivalent to Y397 in Caspase-8A), it failed to interact with the SH2 domain of Src despite the presence of constitutively active (Y527F) Src [15], suggesting that phosphorylation at Y380 of caspase-8B was crucial for its interaction with the SH2 domain of Src. These data together support the hypothesis that phosphorylation of Y397 of caspase-8A gives rise to a phosphotyrosine-containing peptide sequence capable of binding to the Src SH2 domain and promoting Src autophosphorylaton at Y416. These results are also consistent with our finding that Y397E/Y465F exhibits less Src-activating capacity than even the WT; this mutant may act as a dominant negative, which is capable of binding to Src but incapable of activating it.

In accordance with its impact on Src phosphorylation, we also showed that Y465E caspase-8 contributes to the activation of Erk1/2. Several previous observations support the presence and functional role of the inter-relationship between Erk1/2 and caspase-8. Namely, caspase-8-deficient neuroblastoma cells failed to show Erk1/2 activation by fibronectin-dependent adhesion [18]. Likewise, Erk1/2 activation was also absent in these cells upon EGF, PDGF or TNF stimulation [17], while overexpression of caspase-8 led to enhanced TNF induced Erk1/2 activation [63]. Of note, caspase-8 has also been shown to be tyrosine phosphorylated upon (EGF) stimulation [14], [17]. Based on our findings, together with the above-mentioned observations and the fact that Src is a well-known upstream activator of Erk [63], we propose that tyrosine phosphorylation of caspase-8 (as mimicked by the Y465E mutation), may promote Erk activation via facilitating Src phosphorylation.

**Fig 7:**
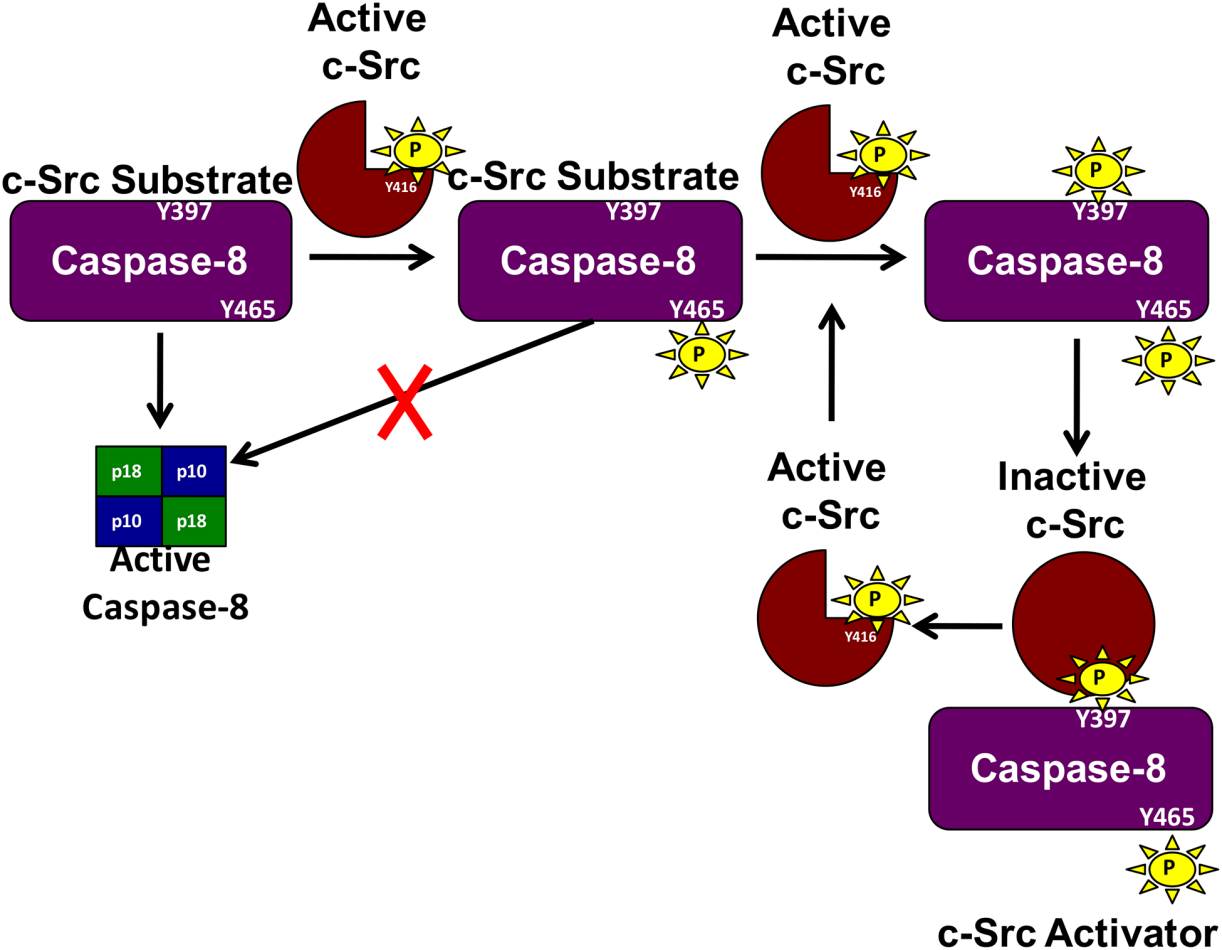
Positive feedback loop of caspase-8A induced Src activation. Phosphorylation of caspase-8A by Src at Y465 prevents its cleavage, and so prevents the induction of apoptosis. The presence of inactive caspase-8A (phosphorylated at Y465) allows the phosphorylation of Y397. When Y397 is phosphorylated in the presence of Y465 phosphorylation, caspase-8A then becomes a Src activator, by binding to the SH2 domain of Src via its pY_397_LEM peptide, further activating Src by promoting Y416 phosphorylation of Src.

We propose that in the presence of a pro-survival signal, caspase-8A becomes phosphorylated at Y465. When Y465 of caspase-8A is phosphorylated, it prevents cleavage and thus casapase-8A activation. Src then phosphorylates caspase-8A at Y397. The phosphorylation of Y397 of caspase-8A provides a phosphotyrosine motif (pY_397_LEM) to interact with SH2 domain of Src and allows Src to assume an open conformation and thus promotes autophosphorylation of Y416 of Src for full activation. This contributes to the suppression of apoptosis and the activation of Src which can induce pro-survival pathways in the setting of a pro-survival environment, acting as a positive feedback loop (Fig 7). Thus, caspase-8 can act both as a substrate and an activator (as an adaptor) of Src, a molecular phenomenon that has been observed with focal adhesion kinase (FAK) [23], AFAP-100 [24] and XB-130 [25].

The regulatory function of casapse-8 might support the dual apoptotic and survival effects of tumor necrosis factor (TNF) [64]. Hughes *et al* demonstrated that when caspase-8 failed to undergo cleavage after binding to death receptor complex (DISC), it failed to induce apoptosis. Our data suggest that if caspase-8A were phosphorylated at Y465, which prevented its cleavage, its recruitment to DISC would not allow it to induce apoptosis. In turn, the uncleavable caspase-8A could then be further phosphorylated by Src at Y397 and thus could further activate Src and potentially propagate pro-survival signaling.

A limitation of our study is that we have not been able to directly confirm phosphorylation of native Y465 by Src. This is due to the technical difficulty whereby the peptide surrounding Y465 is highly hydrophobic, which precluded it from being released from SDS-PAGE gel for sequencing.

In summary, caspase-8 acts as both a Src substrate and Src modulator. When caspase-8 is tyrosine phosphorylated, it not only loses its ability to induce apoptosis, but also gains the ability to promote survival pathways by functioning as a Src activator. This apoptosis/survival functional switch, resulting from the dynamic interplay between Src and caspase-8, results in a regulatory mechanism that can set the balance between these critical functions by integrating pro-survival and pro-apoptotic inputs under normal or pathological conditions.
